# Nanocarbons Reinforcement Effect into Polyethylene Nanocomposites: γ-Ray Attenuation Potential and Hardness Improvement by the Taguchi Method

**DOI:** 10.1021/acsomega.4c10046

**Published:** 2025-01-29

**Authors:** Thais Cardoso Oliveira, Evelyn Alves Nunes Simonetti, Luciana Simone Cividanes

**Affiliations:** † Department of Chemistry, Fundamental Sciences Division, Aeronautics Institute of Technology (ITA), São Jose dos Campos, São Paulo12228-900, Brazil; ‡ Department of Chemistry, Federal Institute of São Paulo (IFSP), São José dos Campos, São Paulo 12223-201, Brazil

## Abstract

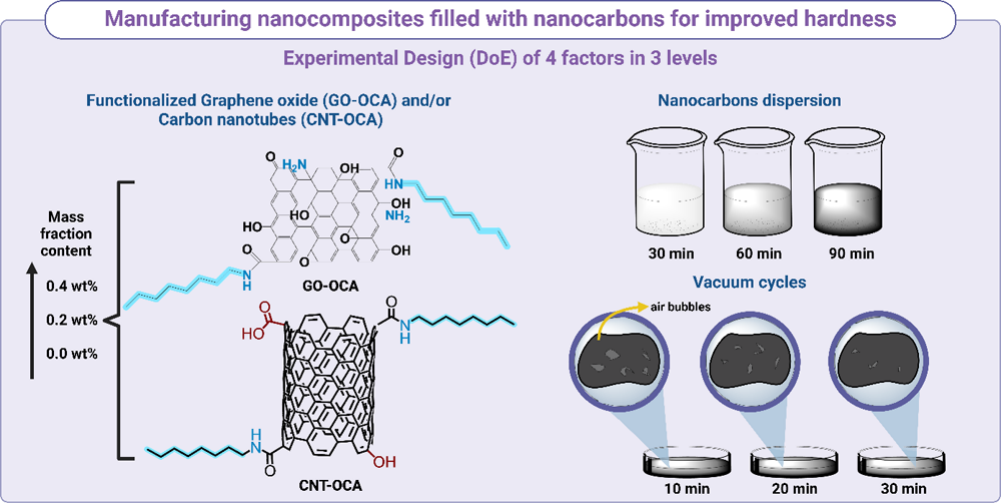

High-density polyethylene (HDPE) is a light and low-cost polymer widely explored as an excellent barrier material for γ-ray and neutron radiation in situations of high exposure (e.g., aerospace). Nevertheless, this polymer is not a suitable structural material due to its low mechanical resistance. Herein, amino-functionalized carbon nanotubes (CNTs) and graphene oxide (GO) were incorporated into HDPE and statistically studied through a Taguchi design of experiments (DoE) model that investigated their synergistic effect. After incorporating these nanocarbons, microscopy images suggest their homogeneous distribution into HDPE. Furthermore, X-ray diffraction shows that the HDPE crystallinity degree increased due to a nucleation effect caused by the nanofillers. Additionally, all nanocomposites presented improved microhardness resistance (up to 69%), particularly when both nanocarbons were incorporated into the matrix. The nanocomposite with higher microhardness resistance had the γ-ray attenuation potential, exhibiting a similar radiation shielding effect to HDPE, with a linear attenuation coefficient higher than Aluminum, a reference material. Hence, this study shows that the synergy between these nanocarbons can be explored to improve HDPE hardness without negatively affecting its attenuation ability. Therefore, these nanocomposites with improved hardness and γ-ray shielding are potential materials for applications requiring surface durability and radiation resistance, such as in air-/spacecraft systems.

## 1 Introduction

Carbon nanotubes (CNTs) and graphene oxide (GO) exhibit remarkable properties such as large surface area, electric/thermal conductivity, and high mechanical resistance.^1−5^ Hence, nanocomposites based on polymer matrices filled with these nanocarbons are widely employed as structural materials due to their increased mechanical properties.^6−8^

As one of the most produced polymers worldwide, high-density polyethylene (HDPE) is a great candidate for developing carbon-based nanocomposites.^9^ This chemically inert polymer, composed of C and H atoms, mainly presents a linear chain with regular packing. Compared to other PE types (e.g., low-density PE), the HDPE exhibits higher stiffness and crystallinity.^10^ Regarding applications, polyethylene is a known γ-ray barrier in the aerospace field, being a reference material for secondary radiation attenuation due to its low tendency to cross-link (slowing the polymer’s degradation).^11,12^

Frequent over exposure to ionizing radiation may cause health problems such as genetic damage and cancer.^13^ Materials for radiation shielding were developed to minimize the harmful effects of the direct exposure to radiation — from the use of Pb-based materials to polymer micro- and nanocomposites.^14^ Hence, due to its H atom content, different kinds of polyethylene-based materials were studied as a lightweight alternative to reference heavy-weight metals for radiation shielding materials (e.g., aluminum and steel).^15^

Because of this polymer attenuation property, polyethylene-based composites were developed with various fillers such as metal oxides and carbon-based nanomaterials.^16^ Moreover, some works focusing on HDPE-based composites exhibited an equivalent or improved attenuation capacity compared to the neat polymer.^17−20^ However, although polyethylene is an excellent material for radiation shielding, this polymer cannot be used as a high-performance structural material due to its low mechanical resistance. In this sense, carbon-based fillers are extensively researched in polymer-based composites for radiation shielding.^16,21−23^

Recently, studies regarding hybrids of CNTs and graphene derivatives demonstrated a synergic effect, resulting in the improvement of the polymer’s electrical conductivity and mechanical properties.^6^ The hybrid nanocarbons improved PE’s tensile strength,^24−26^ electrical conductivity,^27^ surface resistivity,^28^ and thermal stability.^29^ Nonetheless, to enhance these properties, some manufacturing parameters are essential (e.g., nanocarbon dispersion, nanocarbon mass fractions, and the cavitation effect, among others).

Dispersion is a known problem as the nanocarbons’ tendency to agglomerate can decrease the nanocomposite’s mechanical properties instead of improving it.^9,30^ One common approach for the compatibilization of nanofillers such as nanocarbons with polymer matrices is the nanofiller surface modification.^31^ Therefore, the chemical groups introduced in the surface of the nanofiller after its functionalization can be used to tailor the nanofiller’s properties and affinity with previously incompatible materials. Hence, in this work, the CNTs and GO were functionalized with octylamine (OCA) and added to HDPE in different contents. The OCA alkylamine presents a C8-saturated chain, which should improve CNTs and GO compatibility with HDPE, a nonpolar polymer, increasing the chemical affinity between the nanofiller and polymer. Furthermore, the alkyl chain introduced into the nanocarbon surface acts in the separation of the nanocarbon particles through steric hindrance.

Factorial experiments decrease the number of runs to examine significant parameters in experiment.^32^ In this sense, Taguchi experiments are highly fractioned orthogonal arrays typically applied to optimize such manufacturing processes.^33^ Moreover, in the literature, some critical factors (i.e., CNT and GO mass fractions, the nanofillers dispersion time, and the cavitation effect) were not yet studied by a design of experiments (DoE) model.

This work explores the aforementioned manufacturing factors as a function of the nanocomposites’ hardness. Furthermore, the synergistic effect of addition of CNTs and GO into HDPE properties, particularly the γ-ray attenuation, is not yet elucidated. Thus, taking advantage of nanocarbons’ synergistic effect, HDPE reinforced by amino-functionalized CNTs and GO was applied for the first time as a high-energy particle shield material to evaluate its potential as an improved mechanical structural aircraft component with γ-ray shielding properties.

## 2 Experimental Section

### 2.1 Materials

Tetrahydrofuran (THF; 99.5%, Sigma Aldrich) was used without further purification, and the HDPE pellets (IG58, Brasken; 0.956 g cm^–3^) were used as received. The CNT and GO functionalization with OCA (CNT-OCA and GO-OCA) and their characterization were previously described.^31^

### 2.2 Manufacturing of the Nanocomposites Using a Taguchi Orthogonal Array

The nanocomposites were prepared by wet mixing and casting, adapted from previous work.^30^ Briefly, CNT-OCA and/or GO-OCA were dispersed in 20 mL of THF for 30 min by sonication at room temperature. Then, 20 g of HDPE pellets was added to the nanofiller’s dispersion at 70 °C for 30, 60, or 90 min. After the solvent’s evaporation (80 °C for 20 h), the neat HDPE and nanocomposites were prepared by casting; the polymer was melted, mechanically stirred, and cast in an oven at 195 °C under vacuum (five cycles of vacuum at 10, 20, or 30 min).

The samples’ manufacturing followed a Taguchi orthogonal array composed of four factors at three levels arranged in nine runs (Table 1). These studied factors were GO-OCA and CNT-OCA mass fractions (% GO-OCA, factor A; % CNT-OCA, factor B), time under vacuum (*t*_vac_, factor C), and time under dispersion (*t*_disp_, factor D). Regarding the level values, for factor mass fraction, the maximum % in mass (0.6%) was limited due to previous studies from the group;^34,35^ the tendency of both CNTs and GO in agglomerate has a direct impact in the nanocomposite mechanical performance (e.g., hardness and tensile strength). The dispersion factor is also related to the tendency of aggregation of the nanofillers and preliminary results exhibited a time dependency for the nanocarbons dispersion in solvents; the values were chosen considering the total time spent in this step of the process and by qualitative changes in the dispersion levels.^31^ Finally, the times under vacuum were selected considering the total cycles and visual differences regarding the formation of air bubbles from this step; the air bubbles lead to mechanical failure in tests such as microhardness and tensile strength.

**Table 1:** Taguchi Design Matrix: Experimental Run Order Performed of the Samples Prepared Using a Design of Experiments (DoE) with Four Factors at Three Levels (Coded and Actual Values); Nanocarbon Mass Fraction (Factor A, % GO-OCA; Factor B, % CNT-OCA), Time under Vacuum (Factor C, *t*_vac_), and Time under Dispersion (Factor D, *t*_disp_) Set at Low (1), Intermediate (2), and High (3) Levels

run	sample	% GO-OCA	% CNT-OCA	*t*_vac_	*t*_disp_
1	L1	1 (0.0%)	1 (0.0%)	1 (10 min)	1 (30 min)
2	L2	1 (0.0%)	2 (0.2%)	2 (20 min)	2 (60 min)
3	L3	1 (0.0%)	3 (0.4%)	3 (20 min)	3 (90 min)
4	L4	2 (0.2%)	1 (0.0%)	2 (20 min)	3 (90 min)
5	L5	2 (0.2%)	2 (0.2%)	3 (30 min)	1 (30 min)
6	L6	2 (0.2%)	3 (0.4%)	1 (10 min)	2 (60 min)
7	L7	3 (0.4%)	1 (0.0%)	3 (30 min)	2 (60 min)
8	L8	3 (0.4%)	2 (0.2%)	1 (10 min)	3 (90 min)
9	L9	3 (0.4%)	3 (0.4%)	2 (20 min)	1 (30 min)

### 2.3 Physicochemical, Mechanical, and Morphological Characterization

Neat HDPE (L1) and nanocomposites (L2 to L9) morphology were assessed by scanning electron microscopy (FE-SEM), after cryogenic fracture and gold coating, in a JEOL JSM-5310. Then, X-ray diffraction (XRD) patterns from all samples were obtained in a PANalytical Philips X’Pert (Cu(Kα), λ = 1.5406 Å) from 10° ≤ 2θ ≤ 75°; the crystallization degree was calculated as described in previous studies.^30^ Furthermore, samples L1 to L9 were submitted to Rockwell microhardness testing (HRR scale) in a Wilson Hardness Tester from 10 to 60 kgf; the microhardness results were used for the DoE and statistical analysis, which was conducted in Design-Expert and R software. Finally, samples L1 and L6 were characterized by γ-ray spectroscopy equipped with a high-purity germanium radiation detector (HPGe); the measurements used standard ^241^Am (59 keV), ^133^Ba (81 and 383 keV), and ^137^Cs (662 keV) sources that were provided by the International Atomic Energy Agency (IAEA). The theoretical μ/ρ coefficient was determined using the National Institute of Standards and Technology (NIST) XCOM software.^36,37^

## 3 Results and Discussion

### 3.1 Effect of Amino-Functionalized CNTs and/or GO in HDPE Crystallinity Degree

**Figure 1:**
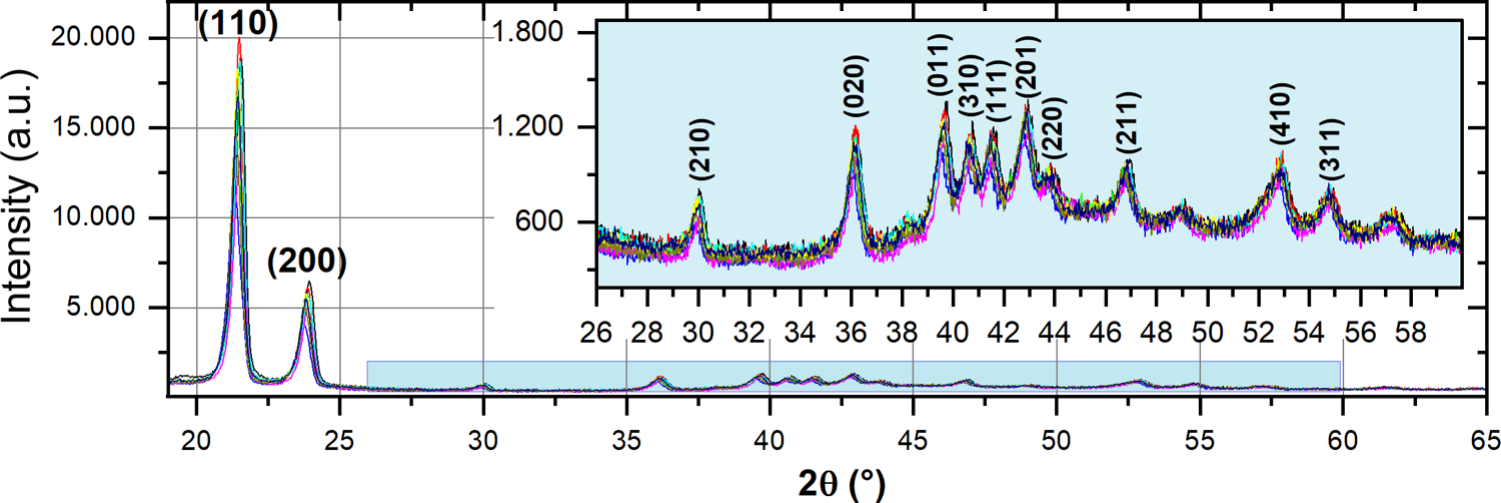
XRD patterns of neat HDPE (L1) and nanocomposites (L2 to L9); according to the attributed peaks, all samples present the characteristic orthorhombic crystalline structure of this polymer. The crystallinity degree calculation used the (110) and (200) peak intensities.

Neat HDPE (L1) and nanocomposites (L2 to L9) had their crystallographic patterns determined (Figure 1). The two prominent peaks at 19° and 25° are attributed to planes (110) and (200), respectively; additionally, peaks at 26° to 55° are assigned in the XRD pattern. All samples showed the typical planes of the HDPE orthorhombic cell unit.^38^

All nanocomposites presented a higher crystallinity degree than neat HPDE (Table 2), ranging from 0.66 to 10.64%. The crystallinity degree increase is attributed to the nanocarbons nucleation effect in HDPE crystallization.^39^ The same nucleation effect is reported in dodecylamine-functionalized CNT-reinforced HDPE nanocomposites, in which a slight increase in the crystallinity degree was observed.^34^ Furthermore, the absence of a plane (002) indicates that the nanocarbons were vertically aligned in the HDPE interior. Additionally, this absence suggests the nanocarbons were effectively dispersed into the matrix, probably due to noncovalent interactions (CH-π) between the nanofiller surface and the matrix, preventing CNT-OCA/GO-OCA agglomeration.^29^

**Table 2:** Samples L1 to L9 Calculated Crystallinity Degree (*W*_c,x_) and the Crystallinity Degree Enhancement Percentage of the Nanocomposites Compared to the Neat HDPE (L1)

	*W*_c,x_ (%)	increase in *W*_c,x_ (%)
L1	65.96	
L2	69.17	4.86
L3	66.39	0.66
L4	66.90	1.43
L5	70.04	6.18
L6	71.61	8.57
L7	72.98	10.64
L8	69.03	4.66
L9	70.27	6.54

### 3.2 Effect of Amino-Functionalized CNTs or GO on HDPE Superficial Mechanical Resistance: Microhardness Maximization Using the Taguchi Method

**Figure 2:**
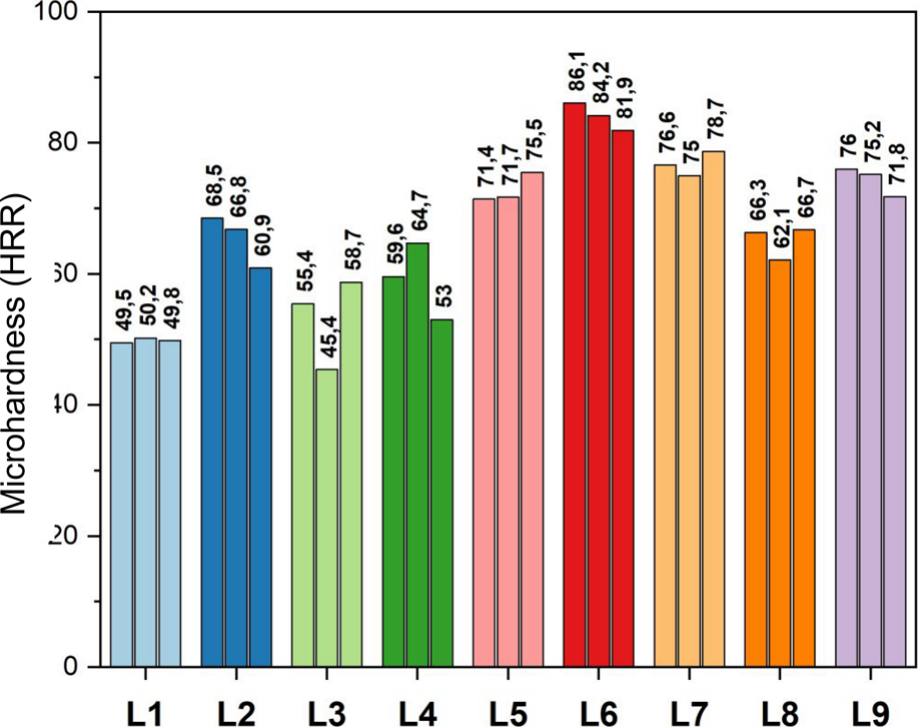
Neat HDPE (L1) and nanocomposites (L2–L9) Rockwell microhardness values obtained in triplicate. The Rockwell microhardness measurement is a comparative test, and the neat HDPE was considered the standard material for microhardness value.

The microhardness values (HRR Rockwell scale) for all nanocomposites (L2 to L9) were higher than for the neat HDPE (L1), representing an increase in the superficial mechanical resistance of 7 to 69% after the incorporation of GO-OCA and/or CNT-OCA (Figure 2 and Table 3). Comparing all samples, sample L6 (0.2% GO-OCA/0.4% CNT-OCA) exhibited a higher increase in microhardness (∼69%). Additionally, the hybrids L5, L8, and L9 displayed higher microhardness values than nanocomposites reinforced only by one nanocarbon, except for sample L7 (0.4% GO-OCA), which presented an increase of ∼54%. The microhardness increase indicates a synergistic effect between CNT and GO. The nanocarbons synergy is due to GO’s high surface area and CNTs’ high aspect ratio, which positively impacts the material’s superficial mechanical resistance.^40^ Moreover, as discussed in the XRD section, the degree of crystallinity is directly related to microhardness. Thus, the increase in crystallinity contributed to the polymer plastic deformation improvement.^41^ Regarding the amino functionalization, the hardness improvement suggested that the nanocarbons were effectively dispersed into the matrix; furthermore, the better distribution of the nanofillers is probably due to the alkyl chain attached to the CNT/GO surface.^34,42^

**Table 3:** Rockwell Microhardness Mean Values of the Neat HDPE (L1) and Nanocomposites (L2–L9); the Microhardness Enhancement Percentage of the Nanocomposites Is Compared to the Neat HDPE (Standard Material)

	microhardness mean (HRR)	increase in microhardness compared to the neat HDPE (%)
L1	49.83	
L2	65.40	+31.24
L3	53.16	+6.68
L4	59.10	+18.59
L5	72.86	+46.22
L6	84.06	+68.69
L7	76.76	+54.04
L8	65.03	+30.50
L9	74.33	+49.16

To optimize the nanocomposites manufacturing, some processing parameters were examined through DoE. Importantly, the Taguchi model was selected considering limited runs as this is a highly fractional design, and the factors were selected considering important nanocarbon-reinforced polymer nanocomposite parameters.^9,30,35^ Factors A and B (nanocarbons % GO-OCA and % CNT-OCA) are related to the nanofiller’s dispersion into the matrix; these nanocarbons’ concentration was studied at two different values. Moreover, a lower mass fraction amount was used as higher concentrations can negatively affect the mechanical resistance due to the nanocarbons agglomeration, forming fracture concentration points.^35^ Another dispersion-related factor studied was the sonication time under dispersion (*t*_disp_). Then, the fourth factor analyzed was the time under vacuum (*t*_vac_) that affects the material’s mechanical integrity; this factor refers to the cavitation and air insufflation that occurs in the mechanical stirring stage, as they can form fragile sites that tend to mechanical failure.

**Figure 3:**
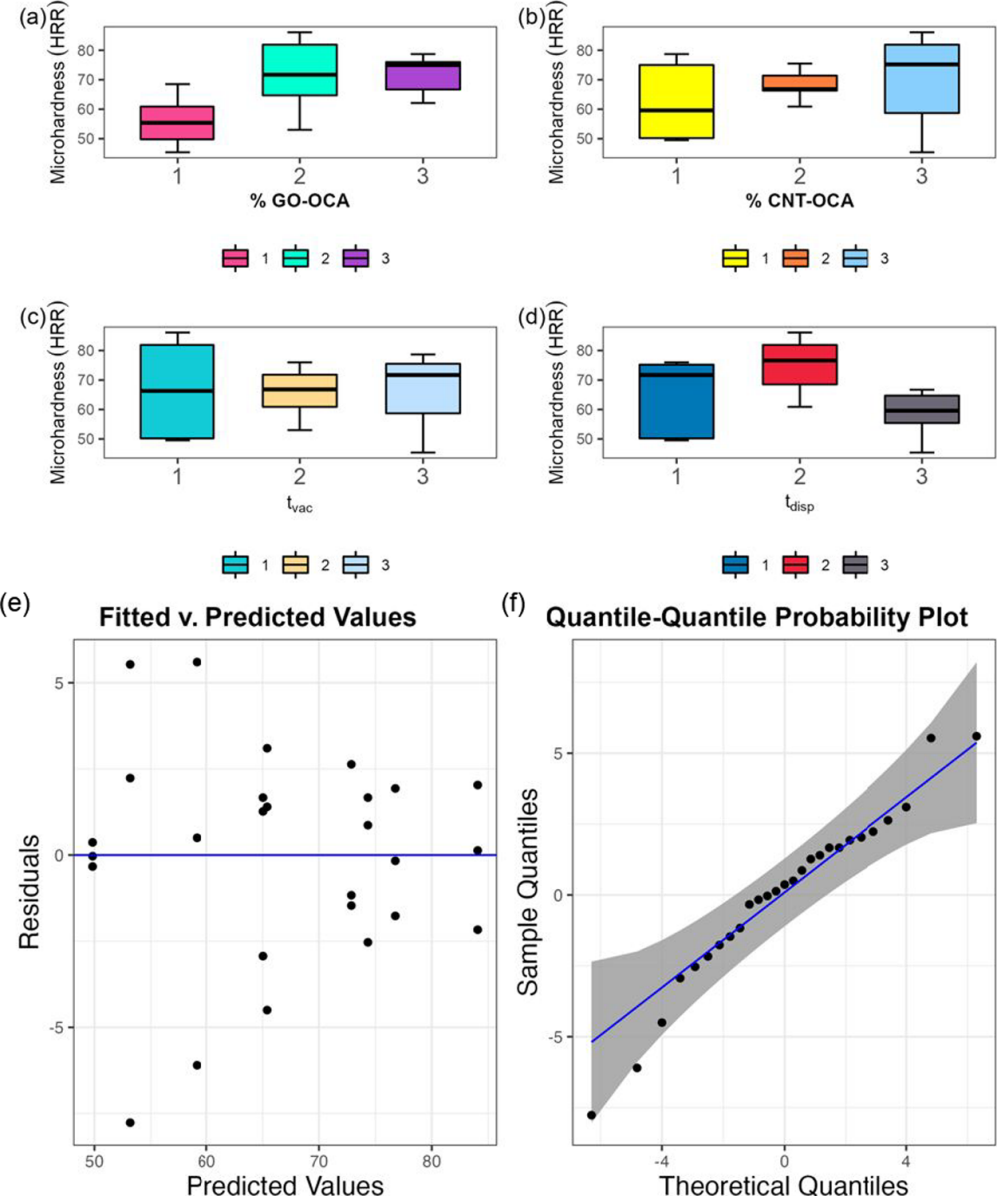
Top: boxplot as a function of samples L1 to L9 microhardness according to the studied four factors at the low (1), intermediate (2), and high (3) levels: nanocarbons mass fraction (a) % GO-OCA and (b) % CNT-OCA; (c) time under vacuum (*t*_vac_); (d) time under dispersion (*t*_disp_). Bottom: statistical model data residual distribution diagnostics: (e) fitted vs predicted values plot; (f) quantile–quantile (Q–Q) normal probability plot. The fitted vs predicted values plot show homoscedasticity, i.e., the variance is the same for the predicted and the actual values, and the residual distribution is normal as no patterns or trends are observed in the Q–Q normal probability plot.

For exploratory analysis, a boxplot was used to assess the data distribution as a function of microhardness (Figure 3); the data distribution does not exhibit outliers. Considering GO-OCA concentration (Figure 3a), the higher microhardness values are obtained when this nanocarbon was added at the intermediary level (0.2%); however, at this level, there is a great variation in microhardness, which is not observed for the higher level (0.4%). Moreover, a different trend is observed for CNT-OCA concentration (Figure 3b): the microhardness value variance is greater at the low and high levels. Thus, even as the highest CNT-OCA concentration was used in samples that exhibited greater microhardness, the intermediary level should produce nanocomposites with minor mechanical resistance variance. Moreover, analyzing the dispersion effect (Figure 3c), intermediate values for this factor have nanocomposites with lower microhardness variance. At last, the intermediary level for factor *t*_vac_ (Figure 3d) also showed more consistency at the microhardness values of the nanocomposites.

The residual diagnostics analyzed through the fitted vs predicted values plot (Figure 3e) and the normal probability plot (Figure 3f) suggests that the data presents homoscedasticity (i.e., the variance is the same for the predicted and actual values) and the distribution is normal (i.e., no evident patterns are observed; information corroborated by the Shapiro–Wilk test, *W* = 0.960, *P* = 0.38).

The ANOVA table (Table 4) indicates that the model is statistically significant (*P* < 0.05), with three of the four studied factors being significant in the material’s processing: factors % GO-OCA (*P* < 0.001), % CNT-OCA (*P* < 0.001), and *t*_vac_ (*P* < 0.001). Nevertheless, despite the nanocarbon distribution importance in the nanocomposite final mechanical properties, *t*_disp_ (*P* < 0.69) was not a statistically significant factor for maximizing the microhardness values.

**Table 4:** ANOVA Table of the Taguchi Experiment: Sum of Squares, Degree of Freedom, Mean Square, *F*-Value, and *p*-Value of the Main Effects (95% Confidence Interval): Nanocarbons Mass Fraction (% GO-OCA and % CNT-OCA), Time under Vacuum (*t*_vac_), and Time under Dispersion (*t*_disp_)

	sum of squares	degree of freedom	mean square	*F*-value	*p*-value
model	3087.27	8	385.91	28.25	<0.001
A: % GO	1515.81	2	757.90	55.47	<0.001
B: % CNT	349.06	2	174.53	12.77	<0.001
C: *t*_disp_	10.23	2	5.12	0.3744	0.692
D: *t*_vac_	1212.17	2	606.09	44.36	<0.001
pure error	245.93	18	13.66		
cor total	3333.20	26			

**Figure 4:**
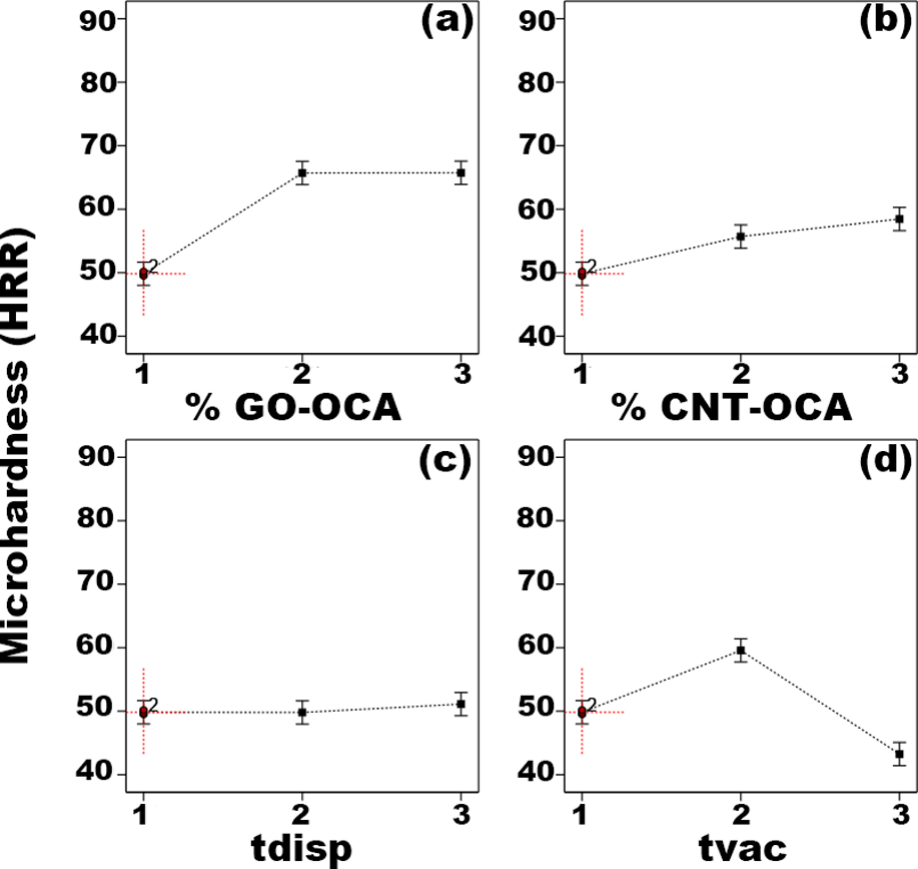
Main effect plot of the four studied factors at low (1), intermediate (2), and high (3) levels as a function of the Rockwell microhardness (HRR): nanocarbons mass fraction (a) % GO-OCA and (b) % CNT-OCA; (c) time under dispersion (*t*_disp_); (d) time under vacuum (*t*_vac_).

Considering the three significant factors, the main effect plot (Figure 4) aids in the determination of the best-studied levels for microhardness maximization. According to the plot, the % GO-OCA (Figure 4a) is similar at intermediate and high levels. Therefore, considering the process cost, the intermediary level is ideal for achieving a greater superficial mechanical resistance. Importantly, the sample with the higher microhardness value (L6) was prepared with the GO-OCA intermediary level. Conversely, for the % CNT-OCA (Figure 4b), the nanocomposites’ microhardness increases with the nanofiller concentration. Thus, to maximize the mechanical resistance, a higher CNT-OCA concentration is the most suitable. Finally, *t*_vac_ (Figure 4d) presented higher microhardness values when set at the intermediary level. Accordingly, the DoE experiment suggests that to maximize HDPE microhardness, factor % CNT-OCA should be used at the high level and % GO-OCA/*t*_vac_ at the intermediary levels, i.e., the nanocomposites produced with 0.2% GO-OCA, 0.4% CNT-OCA, and five cycles of 20 min under vacuum are the ideal parameters to achieve a superior superficial mechanical performance.

### 3.3 Effect of the Amino-Functionalized CNTs and GO Reinforcement in HDPE’s γ-Ray Attenuation

**Figure 5:**
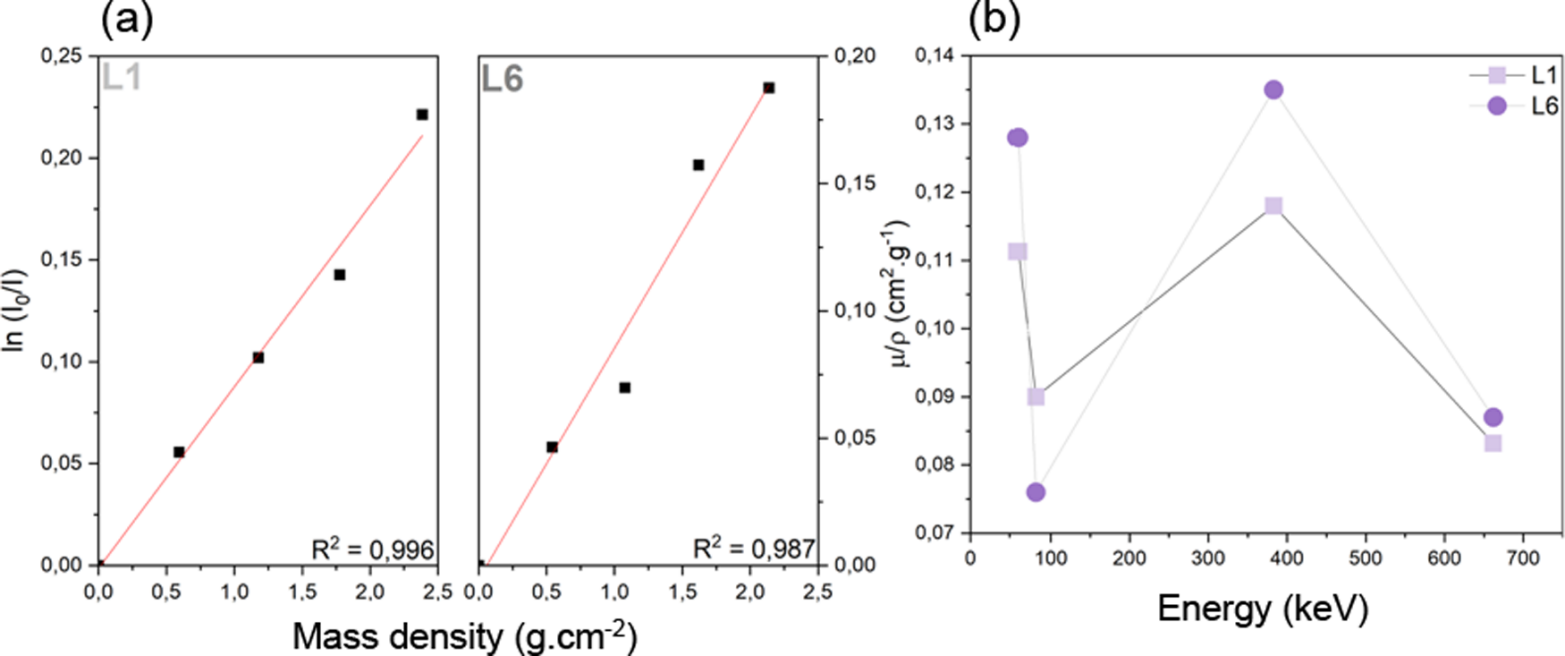
γ-ray spectroscopy measured with ^241^Am, ^133^Ba, and ^137^Cs as radiation sources. (a) Neat HDPE (L1) and L6 nanocomposite ln (*I*_0_/*I*) intensity curve (*I*_0_ = emitted photon energy and *I* = passing through the absorber photon energy) as a function of the absorber’s (sample) mass density; (b) L1 and L6 nanocomposite experimental linear attenuation coefficients as a function of the radiation sources energy.

Apart from suitable mechanical resistance, a structural material for aerospace applications must attenuate the emitted high-energy photons related to the polymeric material degradation, i.e., deterioration due to the formation of peroxide radicals.^43^Figure 5 shows the linear regression of the ln *I*_0_/*I* curve as a function of the sample mass density (*I*_0_ = emitted photon energy and *I* = passing through the absorber photon energy) and the linear attenuation coefficient (μ/ρ) calculated for samples L1 and L6. The experimental μ/ρ values (Table 5) were higher for the nanocomposite (L6) than for HDPE (L1) in three of the four tested emitted energy (^241^Am, ^133^Ba, and ^137^Cs). However, samples L1 and L6 performance as γ-ray shielding materials are considered the same (within the standard deviation; see Table 5). Studies evaluating some nanofillers effect in γ-ray attenuation attribute the shielding effect as a function of the filler mass fraction; at higher nanofiller concentrations, the material’s attenuation performance increases.^12,22,44,45^ CNTs have shown potential in γ-ray attenuation due to their strong interaction with these high-energy photons.^23^ Despite similar μ/ρ values, the nanocomposite exhibited a superior attenuation ability. Therefore, the incorporation of nanofiller into HDPE does not affect the polymer’s known suitability in shielding high-energy particles.

**Table 5:** Neat HDPE (L1) and L6 Nanocomposite Experimental Linear Attenuation Coefficients (μ/ρ), the Experimental Value Standard Deviation, and the Theoretical Linear Attenuation Coefficient (μ/ρ_theoretical_) Obtained Using Different Energy ^241^Am, ^133^Ba, and ^137^Cs as the Radiation Sources

		L1			L6		
	energy (keV)	μ/ρ (cm^–2^ g^–1^)	standard deviation	μ/_ρ theoretical_ (cm^–2^ g^–1^)	μ/ρ (cm^–2^ g^–1^)	standard deviation	μ/ρ_theoretical_ (cm^–2^ g^–1^)
^241^Am	59	0.1113	0.0028	0.1980	0.1280	0.0170	0.1979
^133^Ba	81	0.0900	0.0240	0.1820	0.0760	0.0230	0.1819
	383	0.1800	0.0460	0.1110	0.1350	0.0180	0.1109
^137^Cs	662	0.0832	0.0064	0.0880	0.0870	0.0110	0.0879

Besides comparison of L1 and L6, the μ/ρ values are comparable to the theoretical reference. These two samples presented the expected μ/ρ in high energies (383 keV for ^133^Ba and 662 keV for ^137^Cs); the slight difference in the theoretical and experimental values is owned to the material’s processing as impurities can interfere with the experimental measurement.^46^ Nonetheless, at lower energies (up to 100 keV), the experimental values are below the theoretical ones; this result is attributed to the multiple scattering effects that influence the measurement because the apparatus considers the γ-ray coherent scattering.^47^ Finally, comparing the μ/ρ experimental values to the aluminum μ/ρ theoretical value (Al is one of the reference materials for secondary radiation shielding, presenting μ/ρ = 0.0944 at 383 keV and 0.0747 at 662 keV),^36,37^ the polymer and nanocomposites exhibited higher μ/ρ values (L1, μ/ρ = 0.180 at 383 keV and 0.083 at 662 keV; L6, μ/ρ = 0.135 at 383 keV and 0.087 at 662 keV). Therefore, the nanocomposite demonstrates significant potential as a γ-ray attenuation material.

### 3.4 Morphological Analysis of Amino-Functionalized CNT- and/or GO-Reinforced HDPE

**Figure 6:**
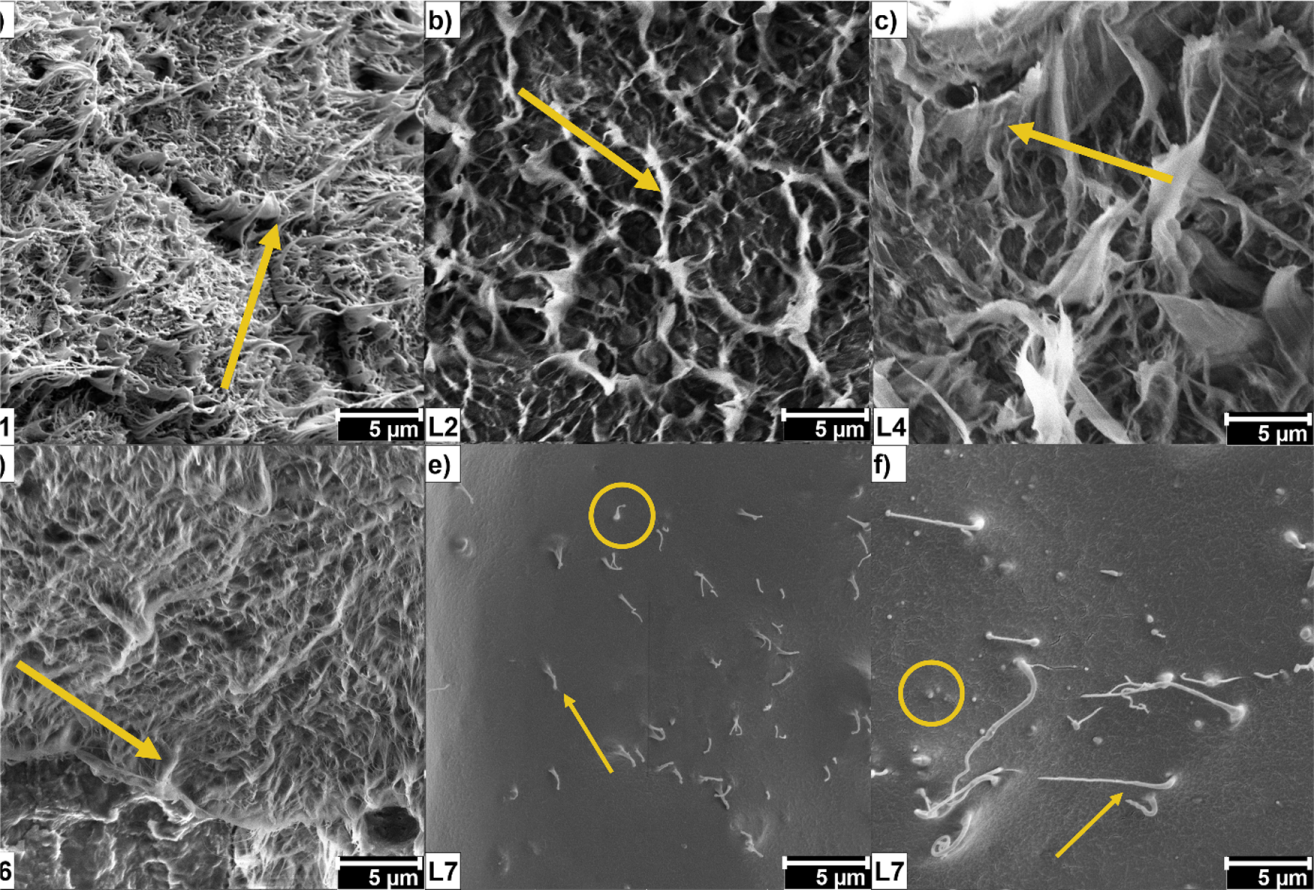
Field-emission scanning electron microscopy (FE-SEM) micrographs of neat (a) HDPE (L1), (b) L2, (c) L4, (d) L6, and (e,f) L7 nanocomposites. Scale bar: 5 μm. (e,f) Different regions in different magnitudes of the same section in sample L7. The filaments (yellow arrows) in the micrographs are typical of HDPE casting at slower cooling rates; the highlighted area in sample L7 (e,f, yellow circle) evidences a thin layer of GO-OCA dispersed into the polymer.

The matrix–nanofiller interface was analyzed through FE-SEM microscopy to verify aggregate formation and irregularities in the nanocomposite. All samples showed similar morphologies (Figure 6a–d). In Figure 6 are represented samples L1 (Figure 6a, neat HDPE), L2 (Figure 6b, 0.2% CNT-OCA/HDPE), L4 (Figure 6c, 0.2% GO-OCA/HDPE), L6 (Figure 6d, 0.2% GO-OCA/04% CNT-OCA/HDPE), and L7 (Figure 6e,f, 0.4% GO-OCA/HDPE). While some authors reported nanocarbons fracture, this affirmation is not possible due to FE-SEM resolution and the nanofiller incorporation into HDPE. The micrographs displayed an entangled structure (yellow arrows), like a fiber mat, which could be attributed to the polymeric chain. This entanglement is mainly related to HDPE films processed by casting and recrystallization between the melting temperature (*T*_m_) and glass transition (*T*_g_).^48^ Therefore, the material casting influenced its morphology, especially considering the polymer crystallization.^49^ Regarding the nanofiller incorporation into HDPE, different sections of the same region were analyzed in sample L7, exhibiting the same “filaments” seen in the neat HDPE sample but in smaller quantities. Comparing both samples, L7 presents a rough texture and some wrinkles in certain regions. Thus, the image suggests that incorporation of GO-OCA into HDPE formed a layer of nanofiller (yellow circles) that coats the polymer fibers. This layer indicates that the material processing efficiently distributed the nanofiller into the matrix, i.e., the nanocarbons are fully incorporated and mixed within the HDPE. Thus, the micrographs corroborate the nanocarbons’ suitable dispersion into the HDPE, which was sufficient to improve the matrix microhardness resistance and γ-ray attenuation.

## 4 Conclusions

Taguchi DoE indicates that the hybrid nanocomposites based on HDPE reinforced by amino-functionalized nanocarbons could be tailored to the required mechanical resistance by adjusting CNTs/GO content and time under vacuum in the casting step. Moreover, compared to neat HDPE, the hybrid nanocomposite with a higher microhardness showed preserved γ-ray shielding properties. Thus, the material exhibits potential as a secondary radiation barrier. Additionally, the nanocomposites can be further improved by increasing the nanocarbons’ mass fraction. Therefore, these hybrid nanocomposites can be used as lightweight structural components in aerospace applications when microhardness resistance is expected and when preserved γ-ray shielding properties are needed.
